# MRI of Hands with Early Rheumatoid Arthritis: Usefulness of Three-Point Dixon Sequences to Quantitatively Assess Disease Activity

**DOI:** 10.5334/jbsr.2692

**Published:** 2022-01-10

**Authors:** Thomas Kirchgesner, Maria Stoenoiu, Patrick Durez, Nicolas Michoux, Bruno Vande Berg

**Affiliations:** 1 Cliniques universitaires Saint-Luc, BE

**Keywords:** rheumatoid, arthritis, MRI, Dixon, hand

## Abstract

The use of efficient treatment with a treat-to-target strategy combined with early detection of the disease completely changed the imaging presentation and outcome of newly diagnosed rheumatoid arthritis (RA) patients. Magnetic Resonance Imaging (MRI) has become the reference technique in clinical research to detect and quantify inflammatory involvement of the soft tissues (synovitis and tenosynovitis) and bone marrow (osteitis) along with structural damages of the bone (erosions) in hands of patients with RA. Three-point Dixon MRI may be a valuable alternative to the currently recommended sequences as it yields effective fat signal suppression, high imaging quality and reproducible assessment of disease activity.

## Introduction

The current article aims to depict the evolution of rheumatoid arthritis (RA) imaging in the last decades and the potential advantages of Dixon Magnetic Resonance Imaging (MRI) sequences in the quantitative assessment of early RA disease activity in hands with the Rheumatoid Arthritis MRI scoring system (RAMRIS).

## Rheumatoid arthritis

RA is a chronic inflammatory disorder affecting multiple organ systems and the most common type of autoimmune arthritis. Between 0.5% and 1.0% of the population suffers from RA worldwide [1]. The physiopathology of RA is complex resulting in synovial membrane inflammation with a predilection for small joints of hands and feet [2]. There are no definitive diagnostic criteria for RA: the final diagnosis is based on the experience of the clinician and the collection of clinical, biological, and sometimes imaging findings. Histology and genetic are not part of the diagnosis of RA in clinical practice. In the absence of effective treatment, RA may result in joint destruction (structural damage) and associated disability. However, the availability of disease-modifying anti-rheumatic drugs (DMARDs), first Methotrexate in the early 1990s and then biological DMARDs in the late 1990s, have dramatically changed the clinical management of RA patients and their outcomes [3].

## Imaging of rheumatoid arthritis

Structural joint damages i.e. bone erosions and cartilage loss reflect chronic active inflammatory involvement of the joint. Historically, plain radiography has been the primary imaging technique to detect, characterize and monitor structural damage in RA patients. Before effective treatment became available, structural damages were common in late RA and presence of bone erosions on radiographs of the hands and wrists was a criterion for the classification of the disease proposed by the American College of Radiology (ACR) in 1987 [4].

New classification criteria including specific autoantibodies (anti-citrullinated protein antibody – ACPA) were developed to improve the detection of early disease. The use of efficient treatment with a treat-to-target strategy combined with early detection of the disease completely changed the radiographical outcome of newly diagnosed RA patients [5,6,7]. Structural damage has become rare and radiographic changes are no longer part of the classification criteria for RA [8].

**Figure 1:**
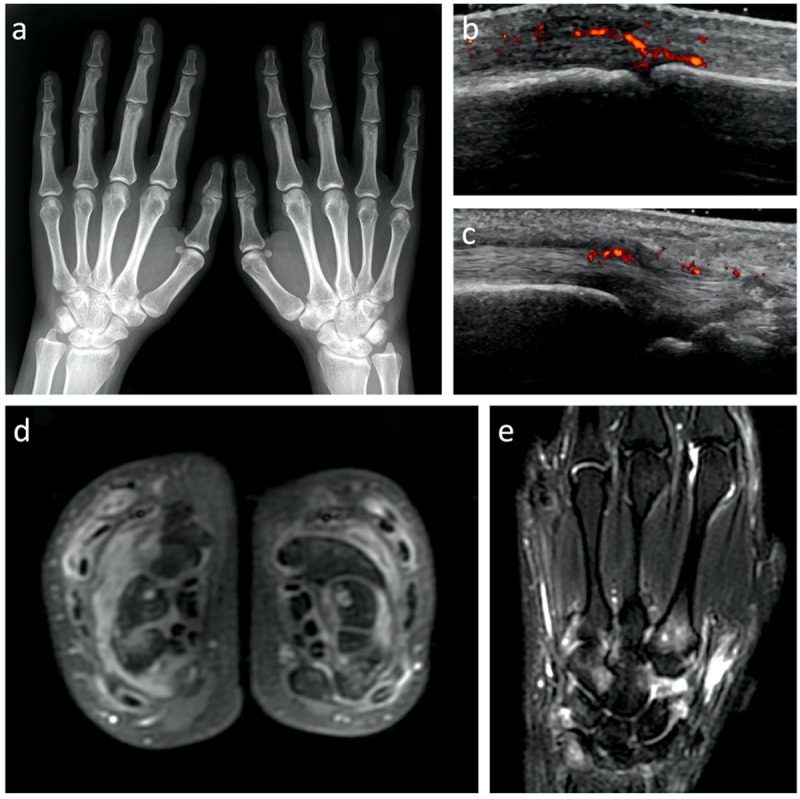
Imaging findings in a 29-year-old female patient diagnosed with early RA including **(a)** radiographs, **(b, c)** Power Doppler ultrasonography and **(d, e)** MRI. While there is no structural damage on radiographs, ultrasonography shows diffuse synovitis (b – synovitis of the 2^nd^ metacarpophalangeal joint of the right hand for example) and tenosynovitis (c – tenosynovitis of the right extensor carpi ulnaris tendon for example). MRI of the hands performed a few days later demonstrates (d) bilateral symmetrical synovitis and tenosynovitis on axial contrast-enhanced fat-suppressed T1-weighted images and (e) osteitis of several carpal and metacarpal bones on coronal fat-suppressed T2-weighted images.

Ultrasonography and MRI emerged as key imaging techniques in the management of RA as both are able to assess early inflammatory involvement of the soft tissues i.e. synovitis and tenosynovitis (***Figure 1***) [9]. Interestingly, MRI is able to assess bone inflammation i.e. osteitis which may serve as a prognostic factor in the outcome of RA [10,11,12,13,14]. In the latest classification criteria, actively diseased joints on ultrasound or MRI can be considered for the active joint count along with clinically active joints [8]. Several studies demonstrated the usefulness of intravenous Gadolinium-based contrast material injection and dynamic analysis of enhancing synovitis on MRI to characterize and monitor the disease [15,16,17]. Contrast-enhanced MRI has been demonstrated more sensitive and specific than non-enhanced fat-suppressed T2-weighted imaging or diffusion-weighted imaging to assess disease activity [18,19]. Accumulation of Gadolinium-based contrast agent in the body is now well established even if its potential toxicity remains uncertain [20,21]. Thus, precautionary measures should encourage the use of alternative to gadolinium-based contrast-enhanced MRI.

Few studies investigated whole-body MRI to assess and monitor RA disease activity in peripheral and axial joints [22,23]. Non-conventional imaging modalities such as 18-F-Fluorodeoxyglucose Positron Emission Tomography (^18^F-FDG PET) and infrared thermography have also been investigated to assess disease activity in RA [24,25,26]. These imaging techniques do not take part in the management of RA in clinical practice.

## Quantitative imaging of rheumatoid arthritis

Methods to quantify disease activity based on medical imaging have been proposed to monitor the disease in clinical practice and establish the efficacy of new treatment in clinical studies.

First several semi-quantitative scoring methods based on bone and cartilage loss on radiographs of the extremities were developed [27]. Among them, the method described by Sharp, later modified by van der Heijde in 1989 still serves as a reference to assess structural damage [28]. Semi-quantitative gradings have also been proposed to evaluate disease activity with grayscale and power Doppler ultrasound. The scoring at ultrasound still shows limited reliability [29,30].

In 2003, Outcome Measures in Rheumatology (OMERACT), a multi-institution study group, developed a semi-quantitative scoring system to asses RA activity at MRI, the RAMRIS [31]. RAMRIS first included the scoring of synovitis, osteitis, and erosions with addition of tenosynovitis and cartilage loss in 2016 [32,33].

To perform the RAMRIS, OMERACT recommends the following MRI ‘core set’ sequences [31,33,34]:

- T1-weighted images in two planes, usually axial and coronal, to assess bone erosions
- fat-suppressed T2-weighted images in one plane, usually coronal, to assess osteitis
- T1-weighted images after intravenous contrast-material injection in the same two planes as before contrast-material injection to assess soft tissue inflammation

An ‘optional’ cartilage dedicated fat-suppressed 3D gradient echo sequence has been proposed to improve the assessment of cartilage [33]. RAMRIS is currently the reference to score disease activity on MRI [35,36,37].

## Fat suppression techniques at MRI

Fat suppression is essential in musculoskeletal MRI as it allows better detection of lesions with increased water content on T2-weighted images and better detection of enhancing tissue on T1-weighted images after intravenous gadolinium-based contrast-material injection. Several techniques are available to obtain fat suppression, and each has its advantages and limits (***Table 1***) [38].

**Table 1:** Advantages and disadvantages of the STIR, CHESS and Dixon fat-suppression techniques in musculoskeletal MRI adapted from [38,54]. PD = Proton Density, SE = Spin Echo, GE = Gradient Echo. Other abbreviations as in the text.


	STIR	CHESS	DIXON

**Mechanism**	Intrinsic fat suppression due to its 180° inversion and 90° excitation pulses	Chemically selective radiofrequency pulse before the acquisition of the signal	Post-processing with addition and subtraction of ‘in-phase’ and ‘out-of-phase’ images

**B_0_ sensitivity**	Insensitive	Sensitive	Insensitive (three-and four-point Dixon)

**B_1_ sensitivity**	Insensitive	Sensitive	Insensitive

**Preferred Field Strength**	Indifferent	High	Medium

**Imaging type**	Not used on T1-weighted images	T1, T2, PD SE, GE	T1, T2, PD SE, GE

**Image quality**	+	+++	+++

**Fat suppression effectiveness**	+++	++	+++

**Imaging time**	Long	Short (depends on the pulse sequence)	Long

**Specific artifacts**	/	Fat suppression failure	Fat-water swapping

**Other**	/	/	Production of four images


Short-Tau Inversion-Recovery (STIR) sequence is insensitive to B0- and B1- fields heterogeneity which therefore brings homogenous fat suppression. However, it is useless on T1-weighted images as it cancels both fat and enhancing tissue and its low signal-to-noise ratio (SNR) is a concern in the evaluation of complex and small anatomical structures as in wrists and hands [38].

The chemical-shift selective (CHESS) technique is based on the frequency-selective presaturation of fat protons. It is commonly used because of its selectivity for fat, high SNR and relatively fast examination time. However, inhomogeneous fat suppression frequently occurs due to its B0- and B1-sensitivity, mostly in anatomical areas with challenging geometric features such as hands.

**Figure 2:**
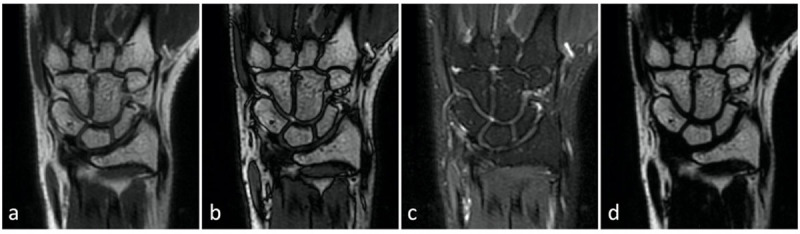
Coronal T2-weighted Dixon images of the right hand in a healthy volunteer. Acquisition of **(a)** in-phase and **(b)** out-of-phase images allows secondary reconstruction of **(c)** fat-suppressed and **(d)** water-suppressed images by post-processing.

The Dixon method was first described in the early 1980s like the CHESS and STIR techniques [39]. It is based on the acquisitions of in-phase and out-of-phase images during the same acquisition with secondary production of ‘water-only’ (i.e. fat-suppressed) and ‘fat-only’ (i.e. water-suppressed) images by post-processing (***Figure 2***) [38]. The acquisition time of Dixon sequences is slightly longer than fat-suppressed sequences using different techniques due to the necessity to acquire the signal at different echo times. Fat-water swapping is an artifact specific to the Dixon sequences. It originates from a natural ambiguity between fat and water peaks which may cause inverted calculation between fat- and water-only voxels and is more frequent using the 2-point than the multi-point (>2) Dixon method [40].

For years, the post-processing time was excessively long and not suitable for use in clinical practice. In the mid 2000s, increased computer performances and other advances in hardware and software allowed drastic reduction of the time needed for post-processing. Since then, interest for the Dixon constantly grew with numerous studies demonstrating its robust homogeneous fat suppression and good image quality in the spine and large joints [41,42,43,44,45].

OMERACT recommendations specifically mention that fat-suppressed T2-weighted images of rheumatoid hands can be obtained either with the CHESS technique i.e. ‘fat saturation’ or with the STIR sequence [31,33]. Fat-suppressed T1-weighted images after contrast-material injection are not specifically recommended by the OMERACT despite its common use in clinical practice and research studies [18,35,46,47,48].

## Dixon sequences in rheumatoid arthritis

We hypothesized that an MRI protocol including three-point Dixon sequences could yield more effective fat suppression and higher image quality than the current recommended sequences while accurately assessing disease activity. We tested the hypothesis that an MRI protocol exclusively based on sequences using the three-point Dixon method was suitable to assess rheumatoid hands and evaluate disease activity according to RAMRIS.

**Figure 3:**
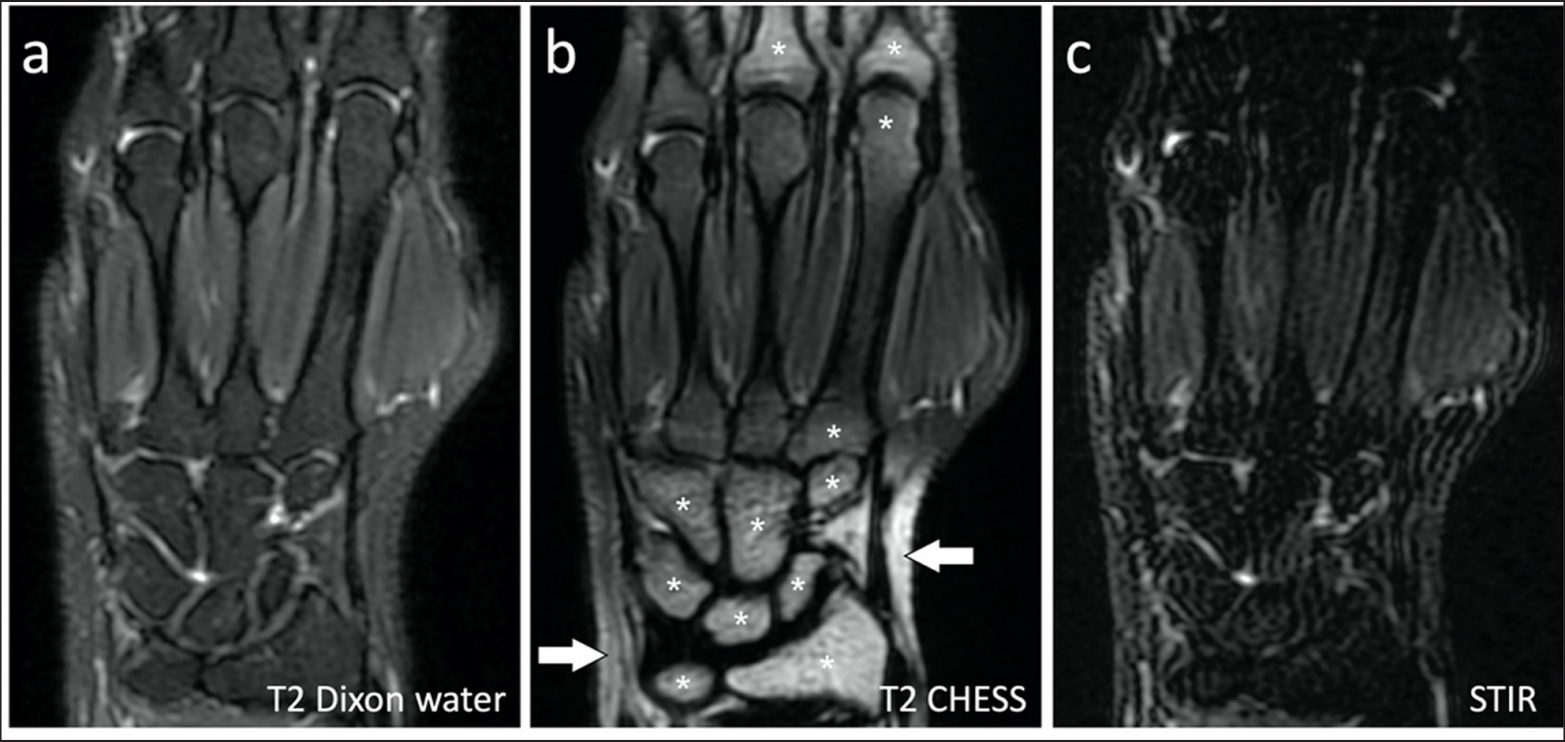
Coronal SE **(a)** T2 Dixon water-only, **(b)** CHESS and **(c)** STIR images of the left hand of the same subject. Fat suppression of bone marrow and soft tissues was effective in all joints with the Dixon and STIR sequences. Fat suppression was ineffective in the bone marrow of several bones (asterisks) and in the soft tissues (arrows) with the CHESS sequence. Note the low image quality of the STIR sequence.

As a first result, our studies consistently demonstrated more robust fat suppression and higher image quality with Dixon- than with CHESS-based MRI protocols to image hands of healthy subjects (***Figure 3***) [49,50] and RA patients at the cost of a longer imaging time [51].

Second, we compared a set of multiple Dixon-based MRI sequences with the recommended set of multiple CHESS-based MRI sequences in a series of 56 hands of patients with suspicion of early RA and demonstrated very good agreement between the two protocols for the assessment of synovitis, tenosynovitis, osteitis and erosions [51].

Then, we compared the scores of disease activity obtained in 48 hands of early RA patients by using either contrast-enhanced T1-weighted Dixon fat- and water-only images or the recommended non-Dixon MRI sequences and demonstrated similar results with the two protocols for the assessment of disease activity suggesting that a short Dixon-based MRI protocol only based on contrast-enhanced T1-weighted images can be used for early RA assessment [52].

**Figure 4:**
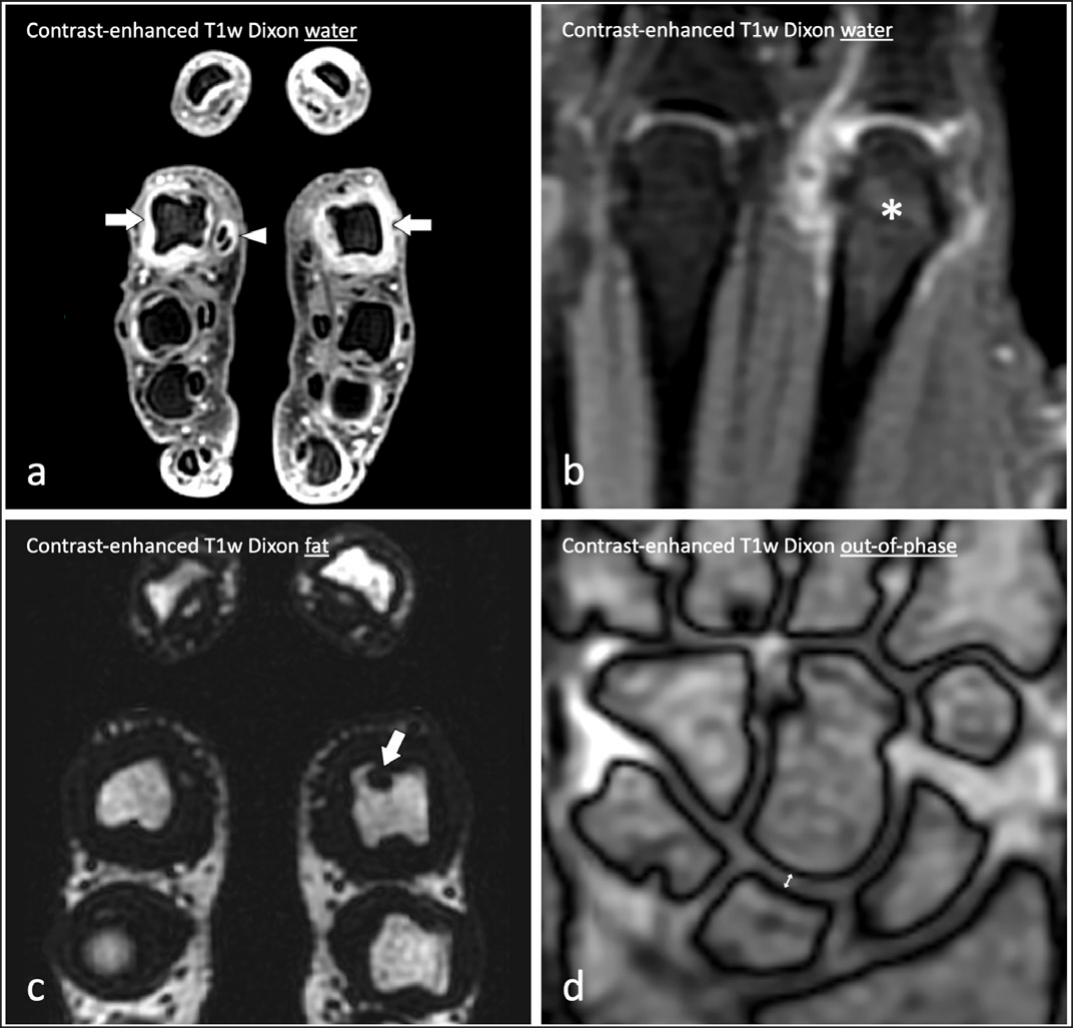
Contrast-enhanced T1-weighted Dixon MRI of the hands of a 28-year-old woman with early rheumatoid arthritis. **(a)** Contrast-enhanced T1-weighted Dixon water-only images in the axial plane demonstrate bilateral synovitis of the second metacarpophalangeal joints (arrows) and tenosynovitis of the flexor digitorum tendons of the right index finger (arrowhead). **(b)** Contrast-enhanced T1-weighted Dixon water-only images in the coronal plane also demonstrate osteitis of the head of the second right metacarpal bone (asterisk) while **(c)** contrast-enhanced T1-weighted Dixon fat-only images in the axial plane demonstrate a bone erosion of the head of the second left metacarpal bone (arrow). In addition, **(d)** contrast-enhanced T1-weighted Dixon out-of-phase images in the coronal plane allow to measure joint space width using India ink artifacts as landmarks and surrogates for the subchondral bone plates (up down arrow in the joint space between the lunate and capitate bones).

Finally, we compared the measurability of hand cartilage using the Dixon sequences in normal subjects and in RA patients. Out of the four available T1-weighted Dixon images, joint-space width measurements performed on Dixon out-of-phase images had the highest correlation coefficient with those on radiographs (***Figure 4***) [53].
